# Association between GDF-15 and sarcopenia progression in older adults: results from a population-based study

**DOI:** 10.1016/j.ebiom.2026.106399

**Published:** 2026-07-17

**Authors:** Yuh-Shiou Gu, Alice Margherita Ornago, Caterina Gregorio, Chiara Ceolin, Anna Picca, Riccardo Calvani, Emanuele Marzetti, Birger C. Forsberg, Davide Liborio Vetrano

**Affiliations:** a Department of Physical Medicine and Rehabilitation, National Cheng Kung University Hospital, Tainan, Taiwan; b Aging Research Center, Department of Neurobiology, Care Sciences and Society, Karolinska Institutet and Stockholm University, Stockholm, Sweden; c Geriatrics Division, Padova University Hospital, Padova, Italy; d Department of Medicine (DIMED), Padova University Hospital, Padova, Italy; e Department of Medicine and Surgery, LUM University, Casamassima, Italy; f Fondazione Policlinico Universitario “Agostino Gemelli” IRCCS, Rome, Italy; g Department of Geriatrics, Orthopedics and Rheumatology, Università Cattolica del Sacro Cuore, Rome, Italy; h Department of Global Public Health, Karolinska Institutet, Stockholm, Sweden; i Stockholm Gerontology Research Center, Stockholm, Sweden

**Keywords:** Growth differentiation factor 15, Trajectory, Biomarker, Disease progression

## Abstract

**Background:**

Growth differentiation factor-15 (GDF-15), a stress-induced cytokine, has been implicated in pathways related to muscle degeneration, although findings regarding its association with sarcopenia remain heterogeneous. This study investigated the association between serum levels of GDF-15 and sarcopenia progression in community-dwelling older adults.

**Methods:**

2347 individuals (mean age 72.3 years [SD 10.4]; 61.6% women), participating in the Swedish National Study on Aging and Care in Kungsholmen, were included in the study. Sarcopenia status (no, probable, and confirmed sarcopenia) were defined according to modified European Working Group on Sarcopenia in Older People 2 criteria. Twelve-year longitudinal sarcopenia trajectories were identified through latent class analysis. GDF-15 was measured in serum samples collected at baseline. Logistic regression was employed to assess the associations between GDF-15 and sarcopenia progression.

**Findings:**

Two trajectories of sarcopenia were identified: an early-progression pattern around the age of 70 and a later-progression pattern around the age of 80. Baseline sarcopenia prevalence differed between trajectories (p < 0.001), and GDF-15 levels were higher in the early trajectory (1.00 ng/mL vs. 0.86 ng/mL, p < 0.001). In adjusted multinomial logistic models, higher GDF-15 was associated with greater odds of probable (aOR = 1.6; 95% CI: 1.2–2.0) and confirmed sarcopenia (aOR = 1.9; 95% CI: 1.3–2.6). GDF-15 levels were also linked to increased odds of belonging to the early progression trajectory (aOR = 1.5; 95% CI: 1.2–1.9), with the association driven by the highest quintiles.

**Interpretation:**

GDF-15 reflects biological processes linked to muscle degeneration and may provide complementary information alongside established measures of sarcopenia in community-dwelling older adults.

**Funding:**

The 10.13039/501100004359Swedish Research Council, the Swedish Ministry of Health and Social Affairs, and the County Councils and Municipalities.

**Research in context**

**Evidence before this study**

Growth differentiation factor 15 is emerging as a key biomarker of ageing, reflecting mitochondrial dysfunction, systemic inflammation, and cellular senescence. It has been associated with physical decline and may contribute to biological pathways implicated in the pathogenesis of sarcopenia. A recent systematic review and meta-analysis reported a small-to-moderate association between higher circulating GDF-15 levels and sarcopenic phenotypes in older adults but also underscored the need for longitudinal studies given the heterogeneity and predominantly cross-sectional nature of available evidence.

**Added value of this study**

In a cohort of community-dwelling older adults, we showed that baseline serum GDF-15 levels are independently associated with both the severity and the longitudinal progression of sarcopenia.

**Implications of all the available evidence**

These findings add to the emerging evidence linking GDF-15 to inflammatory and metabolic processes underlying muscle deterioration. GDF-15 may provide complementary biological information alongside other established measures of muscle performance in the assessment of sarcopenia.

## Introduction

Sarcopenia is an age-related disease, whose early signs appear as early as middle age and accelerate later in life.^1^ The prevalence of sarcopenia in community-dwelling older adults ranges from 10 to 40%, depending on the operational definition employed.^2^ Sarcopenia contributes to a heightened risk of adverse outcomes in older adults, including falls,^3^ fractures,^4^ disability,^5^ and loss of quality of life.^6^

Based on the criteria proposed by the European Working Group on Sarcopenia in Older People 2 (EWGSOP2), sarcopenia is probable when reduced muscle strength is detected. Confirmation of the condition requires additional evidence of low muscle quantity or quality. When impaired physical performance is concurrently present, sarcopenia is classified as severe.^7^ Several factors contribute to the development of sarcopenia, including poor nutrition,^8^ low physical activity levels,^9^ and a range of metabolic and chronic diseases such as cardiovascular diseases,^10^ diabetes mellitus,^11^ cancer,^12^ dementia,^13^ Parkinson's disease,^14^ and multiple sclerosis.^15^ Cellular changes and molecular mechanisms, such as inflammation, oxidative stress, and hormonal changes, also play a role in sarcopenia development.^16^^,^^17^

Growth differentiation factor 15 (GDF-15) is a marker of stress belonging to the transforming growth factor-β superfamily, often referred to as a mitokine due to its regulation by mitochondrial stress. GDF-15 is elevated in sarcopenia and age-related conditions, including cancer, chronic kidney disease, and cardiovascular and metabolic diseases.^18^ In the context of muscle ageing, GDF-15 is increasingly recognised for its multifaceted role. While it primarily serves as a stress-response “messenger” also reflecting mitochondrial dysfunction, emerging evidence indicates that it may also act as a potential mediator when chronically elevated, contributing to the signalling pathways that drive muscle degeneration.^19^^,^^20^ The age-associated impairment of mitochondrial function may lead to the accumulation of reactive oxygen species, which trigger oxidative stress and eventually tissue damage. This stress input activates mitochondrial unfolded protein responses (UPRmt) which leads to increased transcription and secretion of GDF-15.^21^ Thus, the latter may act as a systemic signal that reflects mitochondrial damage, particularly in the skeletal muscle.^21^

To date, studies examining the relationship between GDF-15 levels and sarcopenia in adult and older populations are limited. A recent systematic review and metanalysis reported elevated circulating GDF-15 concentrations among individuals with sarcopenia, with a pooled effect size of moderate magnitude.^22^ However, the robustness of this association is constrained by the small number of included studies, primarily cross-sectional, and the substantial heterogeneity across them. For instance, Kamper et al.^23^ reported higher circulating GDF-15 levels in acutely admitted, frail older inpatients with sarcopenia compared to those without the condition. In contrast, Nga et al.,^24^ focussing on community-dwelling older Korean adults, found no significant difference in serum GDF-15 levels by sarcopenia status. While cross-sectional studies yielded mixed results, longitudinal investigations remain scarce and have not demonstrated a consistent association between GDF-15 levels and incident sarcopenia.^25^^,^^26^ These discrepancies may reflect differences in study populations, settings, follow-up durations, and sarcopenia definitions. Collectively, they underscore the need for further investigation into the relationship between GDF-15 and sarcopenia development and progression in ageing populations.

In the present study, we aimed to explore the association between baseline serum GDF-15 levels and long-term sarcopenia trajectories among community-dwelling older adults.

## Methods

### Study population

We used data from the Swedish National Study on Aging and Care in Kungsholmen (SNAC-K), a prospective population-based study consisting of adults ≥60 years living in Kungsholmen, Stockholm. SNAC-K used randomised, stratified sampling to recruit participants of the following age groups: 60, 66, 72, 78, 81, 84, 87, 90, 93, 96, and 99+ years starting from 2001.^27^ Between March 2001 and June 2004, 3363 of the 4590 eligible individuals participated in the baseline assessment, yielding a response rate of 73.3%. Among the 1227 non-participants, only age groups and sex information were available. Compared with participants, no differences were observed in sex distribution, while age distribution differed slightly (χ^2^ p = 0.035), with a modest overrepresentation of younger individuals among participants. These differences were small and unlikely to affect internal validity; thus, the cohort can be considered broadly representative of the SNAC-K source population. Participants in the oldest age groups (≥78 years) were assessed every 3 years, while those in the youngest groups (age 60–72 years) were assessed every 6 years to analyse age-related changes. The present study used data from the baseline cohort with follow-up information available through December 2016. Given the observational study design and the use of an established population-based cohort, no a priori sample size calculation was performed. Starting from the baseline cohort, we excluded participants with dementia (n = 240), Parkinson's disease or parkinsonism (n = 40), multiple sclerosis (n = 4), and those institutionalised (n = 191). Additionally, 635 participants without GDF-15 measurement and 40 who had incomplete data on sarcopenia were also excluded, obtaining a final study sample of 2347 participants (Supplementary Figure S1). Excluded participants were older, predominantly female, and had a higher number of comorbidities and medications. They also had a higher baseline prevalence of sarcopenia and a more accelerated progression over time (Supplementary Table S1).

The SNAC-K study complies with the principles of the Declaration of Helsinki and was approved by the Regional Ethical Review Board in Stockholm (Dnrs: KI 01–114, 04–929/3, Ö26-2007, 2009/595-32, 2010/447-31/2, 2013/828-31/3, and 2016/730-31/1). Written informed consent to participate in the study was collected from all participants or their next of kin.

### Data collection

Data were collected through structured interviews, clinical examinations, and laboratory tests, conducted by trained nurses, psychologists, and physicians at baseline and at each follow-up visit, adhering to standardised protocols. For participants who were unable to attend the research centre, assessments were carried out through home visits.

### Sarcopenia

Sarcopenia status was defined according to a modified version of the EWGSOP2 criteria.^7^ The methodology for assessing sarcopenia in the SNAC-K cohort has been described previously.^28^^,^^29^ Briefly, the assessment included the evaluation of muscle strength, mass, and physical performance. Muscle strength was assessed using the handgrip strength test or, when handgrip data were unavailable, the chair-stand test. For handgrip strength, low muscle strength was defined as a grip strength <27 kg for men and <16 kg for women.^7^ For the chair-stand test, low muscle strength was defined as a completion time >15 s.^7^ Low muscle mass was defined as a calf circumference below the 20th sex specific percentile (<34 cm for men and <32 cm for women), consistent with previously established cutoffs.^28^ Low physical performance was defined as a walking speed ≤0.8 m/s over 6 m, or 2.4 m for home evaluation, or self-reported slow walking.^28^^,^^29^ According to the EWGSOP2 criteria, participants were classified as follows: non-sarcopenic (normal muscle strength and mass), probable sarcopenic (low muscle strength and normal muscle mass), sarcopenic (low muscle strength and mass), and severely sarcopenic (low muscle strength and mass with physical impairment). Due to the limited number of participants with sarcopenia and severe sarcopenia, these two classes were combined into a single category labelled as confirmed sarcopenia.

### GDF-15 measurement

Peripheral venous blood samples were collected at baseline. Fasting was not compulsory. Serum samples were collected after centrifugation and stored at −80 °C at the Karolinska Institutet BioBank until analysis. Quantification of GDF-15 was carried out at the Affinity Proteomics-Stockholm Unit of the SciLifeLab (Solna, Sweden) using a custom designed Magnetic Luminex Assays—Human Premixed Multi-Analyte assay (Luminex Corporation, Austin, TX, USA–R&D Systems; catalogue LXSAHM-18; lot L146266) and analysed on a FlexMap 3D platform. All samples were processed according to the manufacturer's instructions using a validated standard procedure in 384-well format. Median fluorescence intensity values were acquired using xPONENT software (Luminex), and concentrations were derived using Belysa immunoassay curve fitting software (Millipore). The average intra- and inter-coefficient variations for replicated samples were 16% and 12.3%, respectively. No values were below the assay limit of detection. GDF-15 levels were recorded as continuous variables and subsequently stratified into quintiles.

### Covariates

Sociodemographic covariates included age, sex, body mass index (BMI), functional status, defined by the loss of at least one activity of daily living (ADL), marital status (categorised as partnered or unpartnered), and education level (elementary, high school, university or above). Ethnicity was not examined, as 99% of SNAC-K participants were white Caucasian. Lifestyle behaviours were considered, including smoking habits (never, former, current), alcohol consumption (never, light/moderate, heavy), and physical activity. Based on a self-reported questionnaire, participants were classified as physically active if they reported engaging in light or moderate-to-intense activities several times per week. Chronic diseases were first identified through medical interviews, physical examination, laboratory tests, medication usage, and data from the National Patient Register, and were subsequently coded according to the International Classification of Diseases, 10th revision.^30^ The total number of chronic diseases and daily medications were also recorded.

### Statistical analysis

Baseline characteristics were reported as mean and standard deviation (SD), median and interquartile range (IQR), or count and percentages (%). The assessment of distributional characteristics was based on graphical inspection and pragmatic considerations; given the large sample size, formal normality tests were not solely relied upon. Mean (SD) and parametric tests were used when considered appropriate, robust, or consistent with prior literature. Characteristics were compared between groups through Student's t-test, Kruskal–Wallis test, Wilcoxon rank-sum test, Chi-square test, or Fisher's exact test, as appropriate. When the Kruskal–Wallis test was significant, post-hoc pairwise comparisons were performed using Wilcoxon rank-sum tests with Benjamini-Hochberg correction for multiple testing.

Latent sarcopenia trajectories over 12-year follow-up were developed using growth mixture models (GMMs). GMMs identify distinct latent classes based on similarities in patterns of changes in muscle strength and mass over time. Specifically, threshold mixed models were employed to describe the relationship between each level of the ordinal sarcopenia variable (i.e., no sarcopenia, probable sarcopenia, confirmed sarcopenia and severe sarcopenia) and the underlying latent process. To account for its potential confounding effects, age was used as the time scale of the model. The Bayesian Information Criterion (BIC) and the Lo-Mendell-Rubin (LMR) likelihood ratio test were used to determine the number of latent classes. Different combinations of random effects for the intercept and linear slope were tested, with the variance components either allowed to vary by class or constrained to be equal across classes, which were based on the likelihood ratio test (LRT) results. The variance of the quadratic slope was fixed to zero, assuming no individual variation in quadratic slope change over time. Residual variances were considered homogenous across classes but were allowed to vary by age group. Separate GMMs were estimated for women and men. Participants were assigned to the latent class for which they had the highest posterior probability of membership.

To investigate the association between baseline GDF-15 levels and sarcopenia classes, a multinomial logistic regression model was employed. Additionally, logistic regression was used to assess the association of GDF-15, used both as a continuous variable and stratified into quintiles, and the speed of sarcopenia progression (e.g., membership to the early sarcopenia progression trajectory). All models were adjusted for potential confounders. The baseline model adjustment included age, sex, and education, while the fully adjusted model additionally accounted for lifestyle and clinical factors, including smoking habits, alcohol consumption, physical activity, heart and cerebrovascular diseases, diabetes, chronic kidney diseases, chronic obstructive pulmonary disease (COPD), musculoskeletal diseases, and neoplasms. All regression models were fitted using a complete-case approach for the covariates included in each model. Potential differences by sex and age were evaluated by including interaction terms between GDF-15 and age groups or GDF-15 and sex, in all logistic regression models. The significance of these interaction terms was assessed using the likelihood ratio tests. Stratified analyses were conducted when statistically significant difference was observed (p < 0.05), indicating potential effect modification. Lastly, sensitivity analyses were also conducted to assess the robustness of our findings. Specifically, analyses were repeated after excluding participants with: i) cancer at baseline; ii) cerebrovascular disease at baseline; iii) musculoskeletal diseases at baseline; iv) dementia, Parkinsons's or parkinsonism or multiple sclerosis diagnosed during the 12-year follow-up. A two-tailed p-value <0.05 was considered statistically significant. The analyses were performed using R software (version 4.3.2). This observational analysis is reported following STROBE guidelines.

### Role of the funding source

The funders of the study had no role in study design, data analysis and interpretation, or in the writing and submission of the manuscript.

## Results

**Fig. 1:**
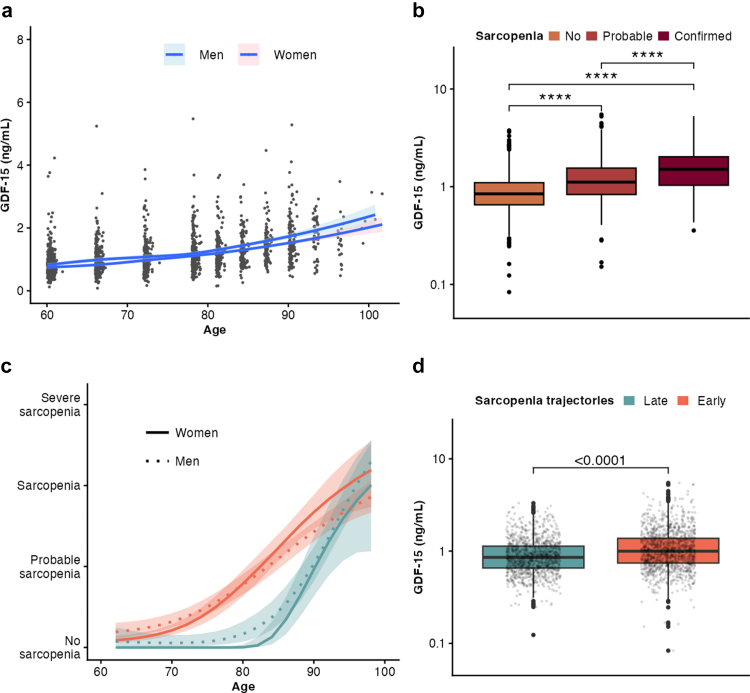
Baseline circulating GDF-15 levels across age, sex and sarcopenia status and progression. Note: a Distribution of baseline GDF-15 across ages by sex. Colour-coded representation of the median (solid line), and 95% confidence interval (shade) for GDF-15 level across ages stratified by sex. b Distribution of GDF-15 across sarcopenia classes at baseline. Boxplots show the median (solid line), the interquartile range (box), and the 2.5th and 97.5th percentiles (whiskers). Overall differences across sarcopenia classes were assessed using the Kruskal–Wallis test. Post-hoc pairwise comparisons were performed using Wilcoxon rank-sum test with Benjamini-Hochberg correction for multiple testing; only significant adjusted p-values are reported (∗∗∗∗: <0.0001). c Colour-coded representation of the median (solid line) and 95% confidential interval (shade) for the late progression and early progression sarcopenia trajectories stratified by sex. The late progression trajectory is depicted in a muted bluish-grey tone; the early progression trajectory in a vivid pink-orange hue. d Baseline distribution of GDF-15 by sarcopenia trajectories. Boxplots show the median (solid line), the interquartile range (box), and the 2.5th and 97.5th percentiles (whiskers). Differences between sarcopenia trajectories were assessed using the Wilcoxon rank-sum test. Abbreviation: GDF15, growth differentiation factor 15.

The mean age of study participants was 72.3 (SD = 10.4) years, and 903 (38.4%) were men. At baseline, 23.1% were classified as probable sarcopenic, and 6.7% as confirmed sarcopenic. As illustrated by Fig. 1a, mean GDF-15 levels increased steadily with advancing age. Across all age groups, men appear to exhibit higher GDF-15 levels compared with women, and the difference between sexes increased with ageing. As displayed in Fig. 1b, GDF-15 levels differed significantly across baseline sarcopenia categories (p < 0.0001). Specifically, GDF-15 increased progressively with worsening sarcopenia stages, with higher levels observed in the probable sarcopenia class compared with no sarcopenia class, and in confirmed sarcopenia class compared with both no sarcopenia and probable sarcopenia classes (all adjusted pairwise comparisons p < 0.0001).

Longitudinal assessment of sarcopenia status, stratified by sex and analysed using growth mixture modelling, revealed two distinct latent trajectories. A total of 1219 participants (51.9%) showed an earlier sarcopenia progression, characterised by an acceleration in sarcopenia status beginning around age 70. In contrast, the late progression trajectory exhibited a marked muscle strength decline starting around age 80 (Fig. 1c). Over time, participants in the early progression trajectory experienced a faster deterioration in sarcopenia status compared to those in the late progression trajectory. Table 1 presents baseline characteristics of the study population overall and according to sarcopenia progression trajectories.

**Table 1:** Baseline characteristics of the study population by sarcopenia trajectories.

	Overall N = 2347	Late progression trajectory N = 1128 (48.1%)	Early progression trajectory N = 1219 (51.9%)	p-value
Age, years, mean (SD)	72.3 (10.4)	70.4 (9.5)	74.1 (10.8)	**<0.001**
Women, n (%)	1445 (61.6)	654 (58.0)	791 (64.9)	**0.0007**
Education, n (%)				**<0.001**
Elementary	349 (14.9)	144 (12.8)	205 (16.8)	
High school	1151 (49.0)	517 (45.8)	634 (52.0)	
University or above	847 (36.1)	467 (41.4)	380 (31.2)	
Partnered, n (%)	1156 (49.3)	632 (56.0)	524 (43.0)	**<0.001**
BMI (kg/m^2^), mean (SD)	25.7 (4.0)	25.9 (3.7)	25.60 (4.3)	0.0827
Chronic diseases				
N° of chronic diseases, mean (SD)	3.7 (2.3)	3.2 (2.0)	4.2 (2.5)	**<0.001**
Hypertension, n (%)	1614 (68.8)	760 (67.4)	854 (70.1)	0.1751
Heart diseases, n (%)	498 (21.2)	189 (16.8)	309 (25.3)	**<0.001**
Atrial fibrillation, n (%)	191 (8.1)	67 (5.9)	124 (10.2)	**<0.001**
Heart failure, n (%)	173 (7.4)	48 (4.3)	125 (10.3)	**<0.001**
Ischaemic heart diseases, n (%)	307 (13.1)	120 (10.6)	187 (15.3)	**0.001**
Cerebrovascular diseases, n (%)	129 (5.5)	41 (3.6)	88 (7.2)	**<0.001**
Chronic kidney diseases, n (%)	750 (32.0)	307 (27.2)	443 (36.3)	**<0.001**
Chronic obstructive pulmonary disease, n (%)	99 (4.2)	36 (3.2)	63 (5.2)	**0.0228**
Diabetes, n (%)	198 (8.4)	76 (6.7)	122 (10.0)	**0.0055**
Musculoskeletal diseases, n (%)	458 (19.5)	192 (17.0)	266 (21.8)	**0.0040**
Inflammatory arthropathies, n (%)	86 (3.7)	35 (3.1)	51 (4.2)	0.1996
Osteoarthrosis, n (%)	303 (12.9)	131 (11.6)	172 (14.1)	0.0818
Other musculoskeletal joint diseases, n (%)	132 (5.6)	47 (4.2)	85 (7.0)	**0.0043**
Neoplasms, n (%)	194 (8.3)	93 (8.2)	101 (8.3)	1.000
N° of drugs, median [IQR]	3 [1–5]	2 [1–4]	3 [1–6]	**<0.001**
1+ ADL lost, n (%)	61 (2.6)	13 (1.2)	48 (3.9)	**<0.001**
Physical and functional status				
Sarcopenia classes at baseline, n (%)				**<0.001**
No sarcopenia	1646 (70.1)	1082 (95.9)	564 (46.3)	
Probable sarcopenia	543 (23.1)	37 (3.3)	506 (41.5)	
Confirmed sarcopenia	158 (6.7)	9 (0.8)	149 (12.2)	
Calf circumference (cm), mean (SD)	36.34 (3.5)	36.70 (3.3)	35.81 (3.7)	**<0.001**
Handgrip strength (kg), mean (SD)	26.6 (11.7)	30.5 (10.9)	22.5 (11.1)	**<0.001**
Impaired handgrip strength, n (%)	471 (20.1)	25 (2.2)	446 (36.6)	**<0.001**
Chair-stand test (sec), median [IQR]	13 [10–20]	12 [9–16]	15 [11–75]	**<0.001**
Impaired chair-stand test, n (%)	888 (37.8)	288 (25.5)	600 (49.2)	**<0.001**
aPhysical limitation, n (%)	406 (17.3)	92 (8.2)	314 (25.8)	**<0.001**
Gait speed (m/sec), mean (SD)	1.06 (0.41)	1.19 (0.34)	0.93 (0.44)	**<0.001**
Impaired gait speed, n (%)	498 (21.2)	115 (10.2)	383 (31.4)	**<0.001**
Physically active, n (%)	1722 (73.4)	899 (79.7)	823 (67.5)	**<0.001**

Abbreviations: SD, standard deviation; IQR, interquartile range; ADL, activity of daily living; BMI, body mass index.

Note: Mismatches between total number and sample size are due to missing data. Missing data for late progression trajectories: BMI = 12; civil status = 1; handgrip strength test = 49; chair-stand test = 2; gait speed = 6; calf circumference = 5. Missing data for early progression trajectories: BMI = 50; civil status = 1; handgrip strength test = 224; chair-stand test = 2; gait speed = 15; calf circumference = 3. All data are presented as mean (SD) or median (IQR) or N (%). p-values represent the comparison between early and late sarcopenia progression trajectories. All statistical tests were two-sided.

a Physical limitation, participants unable to complete chair stand test.

Participants in the early progression trajectory were more likely to be women, older, less educated, unpartnered, physically inactive, and functionally dependent. A significantly higher number of chronic diseases and medications were also observed compared to those in the late progression trajectory. Participants in the early progression trajectory exhibited a significantly advanced sarcopenia status at baseline compared to those in the late progression trajectory (p < 0.001). Specifically, 41.5% of participants in the early progression trajectory had probable sarcopenia and 12.2% had confirmed sarcopenia, compared to 3.3% and 0.8%, respectively, in the late progression trajectory. Baseline GDF-15 levels were significantly elevated among participants in the early progression trajectory compared to late trajectory peers (1.0 vs. 0.86, p < 0.001, Fig. 1d).

Results of the multinomial logistic regression to assess the association between GDF-15 levels and different sarcopenia classes are reported in Table 2. In the baseline model, GDF-15 exhibited a robust association with both probable sarcopenia (OR = 1.7; 95% CI = 1.3–2.1) and confirmed sarcopenia (OR = 2.2; 95% CI = 1.6–2.9). Specifically, each one-unit increase in GDF-15 was associated with 70% and 120% higher odds of being classified as probable or confirmed sarcopenic, respectively. Further adjustments for lifestyle habits and chronic diseases in model 2 led to a slight attenuation of the associations, with a 60% increase in the risk for probable sarcopenia class (OR = 1.6; 95% CI = 1.2–2.0) and a 90% increase in the risk for confirmed sarcopenia class (OR = 1.9; 95% CI = 1.3–2.6), compared with the non-sarcopenic group.

**Table 2:** Association between baseline GDF-15 and baseline sarcopenia classes.

Sarcopenia class	Model 1 OR (95% CI)	Model 2 OR (95% CI)
No sarcopenia	Ref	Ref
Probable sarcopenia	**1.7 (1.3–2.1)**	**1.6 (1.2–2.0)**
Confirmed sarcopenia	**2.2 (1.6–2.9)**	**1.9 (1.3–2.6)**

Odds ratios represent the odds of belonging to the indicated sarcopenia class compared with the non-sarcopenic reference category per one-unit increase in baseline GDF-15 (ng/mL).

Note: Model 1 = adjusted for age, sex, and education. Model 2 = Model 1 adjusted for smoking, alcohol, physical activity, heart diseases, cerebrovascular diseases, diabetes, chronic kidney disease, COPD, MSK diseases, and neoplasms. Model 2 excluded 10 participants due to missing data on smoking and/or alcohol consumption.

Abbreviations: CI, confidential interval; COPD, chronic obstructive pulmonary diseases; GDF-15, growth differentiation factor 15; MSK, musculoskeletal; OR, odds ratio.

Additionally, as shown in Table 3, higher baseline levels of GDF-15 were associated with an increased likelihood of belonging to the early progression trajectory of sarcopenia (OR = 1.7, 95% CI = 1.4–2.1) in the baseline model. This association remained significant, though attenuated, in the fully adjusted model (OR = 1.5, 95% CI = 1.2–1.9). When GDF-15 levels were categorised into quintiles, only participants in the fourth and fifth quintiles showed a significantly higher risk of earlier sarcopenia progression (Table 3).

**Table 3:** Association between baseline GDF-15 and the early progression trajectory of sarcopenia.

GDF-15 (ng/mL)	Model 1 OR (95% CI)	Model 2 OR (95% CI)
Continuous	1.7 (1.4–2.1)	1.5 (1.2–1.9)
1st quintiles	Ref.	Ref.
2nd quintiles	1.07 (0.83–1.39)	1.05 (0.81–1.38)
3rd quintiles	1.08 (0.83–1.41)	1.06 (0.81–1.39)
4th quintiles	**1.48 (1.12–1.94)**	**1.33 (1.01–1.77)**
5th quintiles	**1.93 (1.42–2.62)**	**1.61 (1.16–2.23)**

Note: Model 1: adjusted for age, sex, and education. Model 2: Model 1 adjusted for smoking, alcohol, physical activity, heart diseases, cerebrovascular diseases, diabetes, chronic kidney disease, COPD, MSK diseases, and neoplasms. Model 2 excluded 10 participants due to missing data on smoking and/or alcohol consumption.

Abbreviations: CI, confidential interval; COPD, chronic obstructive pulmonary diseases; GDF-15, growth differentiation factor 15; MSK, musculoskeletal; OR, odds ratio.

Across all models, the inclusion of an interaction term between GDF-15 and sex or GDF-15 and age did not significantly improve model fit (all p-values >0.05), suggesting no evidence of effect modification by sex or age. Therefore, stratified analyses were not conducted.

Sensitivity analyses excluding participants with either cancer or cerebrovascular disease at baseline yielded results consistent with the main analysis (Supplementary Tables S2 and S3 & S5 and S6), while the exclusion of those with baseline musculoskeletal diseases led to an attenuation of the associations (Supplementary Tables S4 and S7). Additionally, the exclusion of participants who developed incident dementia, Parkinson's or parkinsonism or multiple sclerosis during the follow-up yielded comparable estimates (data not shown).

## Discussion

Our findings suggest that baseline serum GDF-15 levels are associated with both the severity and the rate of progression of sarcopenia in older adults. Participants with confirmed sarcopenia had higher GDF-15 concentrations at baseline, and those with greater levels of this cytokine were more likely to follow an accelerated trajectory of sarcopenia over the follow-up. Although these associations were partially attenuated after adjusting for lifestyle and clinical covariates, they remained statistically significant.

GDF-15 is upregulated in the setting of impaired muscle fibre quality, which may contribute to the development of sarcopenia. In line with this, increased GDF-15 expression appears to reflect stress-response pathways activated by age-related mitochondrial dysfunction, linking impaired muscle fibre quality to downstream catabolic signalling.^21^ A mechanism linking chronic GDF-15 elevation with mitochondrial dysfunction and muscle fibre atrophy has been proposed.^31^ It is important to note that autophagy plays a dual role in muscle health; while basal autophagic flux is essential for protein quality control and regeneration, its pathological over-activation, potentially mediated by GDF-15 through caspase-3 and the autophagy-lysosome pathway, can accelerate protein degradation and lead to muscle fibre atrophy. This suggests that GDF-15 may transition from a protective signal to a driver of catabolic changes when its levels exceed a certain physiological threshold. This results in a preferential loss of type II muscle fibres (glycolytic fast-twitch and high force generating muscle fibres), parallelled by reductions in maximal isometric contractile force.^31^ Previous studies have produced mixed results regarding the association between GDF-15 and sarcopenia in community-dwelling older adults. While several have identified cross–sectional associations, evidence from longitudinal studies remains scarce.^22^ For instance, data from the Korean Frailty and Aging Cohort Study showed higher GDF-15 levels among sarcopenic individuals, but no association with incident sarcopenia over a two-year period.^26^ In line with their cross-sectional finding, we also observed higher GDF-15 levels in participants with sarcopenia. However, our extended 12-year follow-up revealed a significant association between GDF-15 and an earlier sarcopenia progression. In contrast, a secondary longitudinal analysis of the Multidomain Approach for Preventing Alzheimer's Disease (MAPT) study reported no association between serum GDF-15 and either the incidence of confirmed sarcopenia or the evolution of its key determinants (i.e., appendicular lean mass, grip strength, and gait speed) over two years.^25^ These discrepancies are likely due to variation in study design, population profiles, follow-up duration, and diagnostic criteria. In particular, distinct diagnostic criteria may influence sarcopenia classification, thereby affecting the comparability of findings across studies. Moreover, shorter follow-up period may fail to capture the gradual progression of sarcopenia. Supporting this, Trevisan et al.^28^ found that most participants remained in their baseline sarcopenia class after one year, especially those classified as non-sarcopenic or probable sarcopenic. However, after 10 years, 40.4% of non-sarcopenic participants and 14.5% of those with probable sarcopenia progressed to more advanced sarcopenia stages.^28^ This initial slow progression of sarcopenia, also observed in our study, suggests that long-term follow-up is essential to detect meaningful changes in sarcopenia progression over time.

Our finding that elevated baseline GDF-15 levels were associated with a faster progression in sarcopenia status supports its involvement in processes driving sarcopenia trajectories among older adults. A hormetic dual role has been proposed for GDF-15 in skeletal muscle.^20^ At moderate levels, GDF-15 may exert protective effects by signalling acute stress responses. While initially considered a compensatory mechanism, its chronic overexpression, as seen during ageing, has been associated with maladaptive effects, including low-grade inflammation and muscle wasting.^20^ This dual behaviour is consistent with our findings that only the highest GDF-15 quintiles were associated with an earlier trajectory of sarcopenia. In this context, GDF-15 has been explored as a potential therapeutic target, particularly in conditions characterised by severe muscle wasting. While its application in sarcopenia is still speculative, clinical trials investigating GDF-15 neutralising antibodies (e.g., ponsegromab) in cancer cachexia have shown promising results in improving body weight and physical activity.^32^^,^^33^ These developments suggest that modulating the GDF-15/GFRAL axis might eventually offer a novel strategy to mitigate the accelerated muscle loss, although its role in sarcopenia and high risk older populations remains to be established.

Our study has several limitations. First, the SNAC-K cohort consists of older adults living in an urban district of Stockholm who are relatively healthy and well-educated, potentially limiting the generalisability of our findings. Additionally, missing data led to the exclusion of over 1000 participants, who were older, had a higher burden of multimorbidity and exhibited more severe sarcopenia and higher GDF-15 levels, which may have resulted in an underestimation of the association examined. Second, muscle mass was estimated using calf circumference, which is less accurate than body composition techniques, such as bioelectric impedance analysis and dual-energy X-ray absorptiometry. However, calf circumference remains an acceptable surrogate of muscle mass in settings where more advanced methods are unavailable.^7^ Lastly, the absence of repeated GDF-15 measurements precluded a dynamic assessment of their association with sarcopenia progression. Despite these limitations, our study provides valuable insights into the evolution of sarcopenia status in relation to GDF-15 level, alongside demographic, lifestyle, and clinical factors. This information may help contextualise sarcopenia within its broader clinical and biological determinants in older adults.

In conclusion, higher GDF-15 levels are associated with more severe sarcopenia status at baseline and with accelerated progression over time. These findings align with previous evidence linking GDF-15 to processes associated with muscle deterioration in older adults. By reflecting inflammatory and metabolic pathways, GDF-15 may provide complementary biological information alongside established measures of muscle performance in the assessment of sarcopenia. Further longitudinal studies with extended follow-up periods are needed to better clarify the role of GDF-15 in the early identification and progression of sarcopenia in older adults. Such studies should also address the substantial challenges involved in establishing clinically meaningful cut-off values for GDF-15, including biological variability, population heterogeneity, and the influence of comorbid conditions. In this context, future research should explore the potential of GDF-15 as a complementary biomarker within a multidimensional sarcopenia assessment framework and evaluate the applicability of our findings across different settings and ethnic groups. Additionally, the GDF-15/GFRAL axis may remain of interest for future therapeutic investigation in both cachexia and sarcopenia.

## Contributors

Conceptualisation: Davide Liborio Vetrano, Caterina Gregorio and Alice Margherita Ornago. Methodology: Caterina Gregorio, and Alice Margherita Ornago. Formal Analysis: Caterina Gregorio and Alice Margherita Ornago. Writing the original draft: Yuh-Shiou Gu. Writing review and editing: Yuh-Shiou Gu, Alice Margherita Ornago, Caterina Gregorio, Chiara Ceolin, Anna Picca, Riccardo Calvani, Emanuele Marzetti, Birger Forsberg, and Davide Liborio Vetrano. Visualisation: Alice Margherita Ornago and Caterina Gregorio. Access and verification of data: Davide Liborio Vetrano, Caterina Gregorio, and Alice Margherita Ornago. All authors have read and approved the final version of the manuscript.

## Data sharing statement

Data are from the SNAC-K project, a population-based study on ageing and dementia (http://www.snac-k.se/). Access to original data is available to the research community on approval by the SNAC-K organisation. Applications for accessing data can be submitted through http://www.snac-k.se/.

## Declaration of interests

All authors declare no conflict of interest.
