# Supplementary material for: Association between GDF-15 and sarcopenia progression in older adults: results from a population-based study
Source: eBioMedicine. 2026 Jul 17;130:106399. doi: 10.1016/j.ebiom.2026.106399 (PMC13400763; doi:10.1016/j.ebiom.2026.106399)
Supplement: Supplementary Material [file mmc1.pdf]

SUPPLEMENTARY MATERIALS

Supplementary Figures

Supplementary Figure 1. Study population flow-chart

Supplementary Tables

Supplementary Table 1. Baseline characteristics of SNAC-K population by exclusion criteria
Supplementary Table 2. Association between GDF-15 and sarcopenia status at baseline excluding participants with neoplasms at baseline (n = 194)
Supplementary Table 3. Association between GDF-15 and sarcopenia status at baseline excluding participants with cerebrovascular diseases at baseline (n = 129)
Supplementary Table 4. Association between GDF-15 and sarcopenia status at baseline excluding participants with musculoskeletal diseases at baseline (n = 458)
Supplementary Table 5. Association between GDF-15 and risk of belonging to the early progression trajectory of sarcopenia excluding participants with neoplasms at baseline (n = 194)
Supplementary Table 6. Association between GDF-15 and risk of belonging to the early progression trajectory of sarcopenia excluding participants with cerebrovascular diseases at baseline (n = 129)
Supplementary Table 7. Association between GDF-15 and risk of belonging to the early progression trajectory of sarcopenia excluding participants with musculoskeletal diseases at baseline (n = 458)

Supplementary Figure 1. **Study population flow-chart**

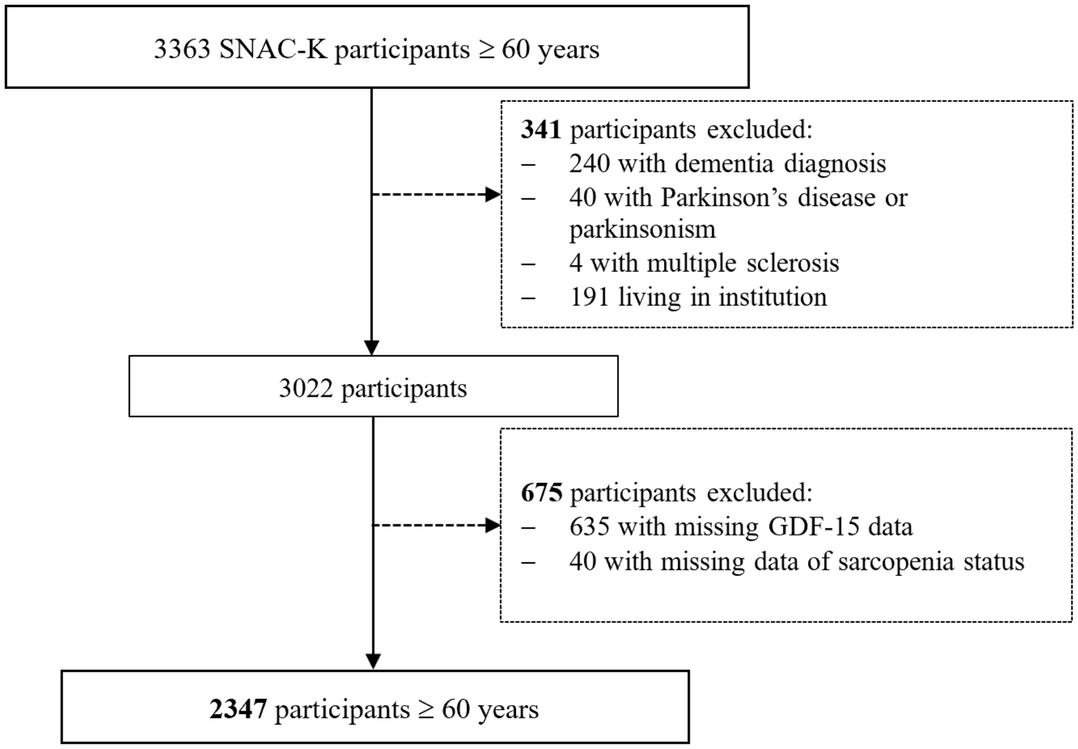

Supplementary Table 1. Baseline characteristics of SNAC-K population by exclusion criteria

	**Overall**<br>**3363**	**Included**<br>**2347 (69.8%)**	**Excluded**<br>**1016 (30.2%)**	**p-value**
Age, years, mean (SD)	74.71 (11.20)	72.31 (10.37)	80.25 (11.07)	**<0.001**
Women, n (%)	327 (9.8)	61 (2.6)	266 (26.4)	**<0.001**
BMI (kg/m^2), mean (SD)	25.56 (4.16)	25.74 (4.02)	25.02 (4.51)	**<0.001**
Education, n (%)				**<0.001**
Elementary	590 (17.7)	349 (14.9)	241 (24.5)	
High school	1651 (49.6)	1151 (49.0)	500 (50.8)	
University	1090 (32.7)	847 (36.1)	243 (24.7)	
Partnered, n (%)	1521 (45.4)	1156 (49.3)	365 (36.4)	**<0.001**
**Chronic diseases**				
N° of chronic diseases, mean (SD)	4.06 (2.48)	3.71 (2.31)	4.86 (2.66)	**<0.001**
Hypertension, n (%)	2277 (67.7)	1614 (68.8)	663 (65.3)	**0.0500**
Heart diseases, n (%)	837 (24.9)	498 (21.2)	339 (33.4)	**<0.001**
Atrial fibrillation, n (%)	324 (9.6)	191 (8.1)	133 (13.1)	**<0.001**
Heart failure, n (%)	353 (10.5)	173 (7.4)	180 (17.7)	**<0.001**
Ischemic heart diseases, n (%)	514 (15.3)	307 (13.1)	207 (20.4)	**<0.001**
Cerebrovascular diseases, n (%)	265 (7.9)	129 (5.5)	136 (13.4)	**<0.001**
Chronic kidney diseases, n (%)	1117 (33.2)	750 (32.0)	367 (36.1)	**0.0206**
Chronic obstructive pulmonary disease, n (%)	167 (5.0)	99 (4.2)	68 (6.7)	**0.0032**
Diabetes, n (%)	296 (8.8)	198 (8.4)	98 (9.6)	0.2845
Dyslipidemia, n (%)	1558 (46.3)	1196 (51.0)	362 (35.6)	**<0.001**
Depression, n (%)	310 (9.2)	188 (8.0)	122 (12.0)	**<0.001**
Musculoskeletal diseases, n (%)	688 (20.5)	458 (19.5)	230 (22.6)	**0.0439**
Inflammatory arthropathies, n (%)	136 (4.0)	86 (3.7)	50 (4.9)	0.1087
Osteoarthrosis, n (%)	425 (12.6)	303 (12.9)	122 (12.0)	0.5051
Other musculoskeletal joint diseases, n (%)	222 (6.6)	132 (5.6)	90 (8.9)	**0.0007**
Neoplasms	299 (8.9)	194 (8.3)	105 (10.3)	0.0615
N° of drugs, median [IQR]	3 [1-6]	3 [1-5]	5 [2-7]	**<0.001**
1+ ADL lost	2182 (64.9)	1445 (61.6)	737 (72.5)	**<0.001**
**Physical and functional status**				
Sarcopenia classes at baseline, n (%)				**<0.001**
No sarcopenia	2036 (63.2)	1646 (70.1)	390 (44.7)	
Probable sarcopenia	869 (27.0)	543 (23.1)	326 (37.4)	
Confirmed sarcopenia	314 (9.8)	158 (6.7)	156 (17.9)	
Early sarcopenia progression trajectory, n (%)	1603 (53.8)	1219 (51.9)	384 (60.5)	**<0.001**
Calf circumference (cm), mean (SD)	35.66 (3.90)	36.24 (3.54)	34.26 (4.37)	**<0.001**
Handgrip strength (kg), mean (SD)	26.06 (11.61)	26.63 (11.68)	23.83 (11.06)	**<0.001**
Impaired handgrip strength, n (%)	636 (24.4)	471 (22.7)	165 (31.0)	**<0.001**
Chair-stand test (sec), median [IQR]	14 [11-75]	13 [10-20]	23 [12-75]	**<0.001**
Impaired chair-stand test, n (%)	1469 (45.1)	888 (37.9)	581 (63.4)	**<0.001**
Physical limitation, n (%)	828 (25.4)	406 (17.3)	422 (46.0)	**<0.001**
Gait speed (m/sec), median [IQR]	1 [0.61-1.20]	1.20 [0.81-1.50]	0.67 [0.22-1.20]	**<0.001**
Impaired gait speed, n (%)	970 (29.9)	498 (21.4)	472 (51.6)	**<0.001**
Physically active, n (%)	2200 (65.4)	1722 (73.4)	478 (47.0)	**<0.001**
**Blood-based biomarker**				
GDF15 (ng/ml), median (IQR)	0.93 [0.70, 1.29]	0.91 [0.69, 1.25]	1.36 [1.00, 1.86]	**<0.001**

SD = standard deviation; IQR = interquartile range; ADL = activity of daily living; BMI = body mass index; GDF15 = growth differentiation factor 15.

Mismatches between total number and sample size are due to missing data. Missing data for included: BMI = 62; civil status = 2; handgrip strength test = 273; chair-stand test = 4; gait speed = 21; calf circumference = 8. Missing data for excluded: education = 32; ADL = 10; BMI = 258; civil status = 13; dementia = 10; sarcopenia status at baseline = 144; sarcopenia latent profile = 381; handgrip strength test = 484; chair-stand test = 99; gait speed = 101; calf circumference = 59; GDF15 = 872.

All variables are presented as mean (SD) or median (IQR) or N (%). *p-values* represent comparison between included and excluded participants. All the statistical tests were two-sided.

Supplementary Table 2. Association between GDF-15 and sarcopenia status at baseline excluding participants with neoplasms at baseline (n = 194)

GDF-15 (ng/mL)	Model 1<br>OR (95% CI)	p-value	Model 2<br>OR (95% CI)	p-value
Non sarcopenic	Ref	-	Ref	-
Probable sarcopenic	**1.6 (1.3-2.0)**	**<0.001**	**1.5 (1.2-1.9)**	**0.0016**
Sarcopenic	**2.0 (1.5-2.8)**	**<0.001**	**1.8 (1.2-2.5)**	**0.0019**

Model 1: adjusted for age, sex, and education.
Model 2: model 1 adjusted for smoking, alcohol, physical activity, heart disease, cerebrovascular diseases, diabetes, CKD, MSK diseases, and COPD. Model 2 excluded 9 participants due to missing data on smoking and/or alcohol consumption.
Abbreviations: CI = confidential interval; COPD = chronic obstructive pulmonary disease; CKD = chronic kidney disease; GDF-15 = growth differentiation factor 15; MSK = musculoskeletal; OR = odds ratio.

Supplementary Table 3. Association between GDF-15 and sarcopenia status at baseline excluding participants with cerebrovascular diseases at baseline (n = 129)

GDF-15 (ng/mL)	Model 1<br>OR (95% CI)	p-value	Model 2<br>OR (95% CI)	p-value
Non sarcopenic	Ref	-	Ref	-
Probable sarcopenic	**1.6 (1.3-2.1)**	**<0.001**	**1.5 (1.2-1.9)**	**0.0011**
Sarcopenic	**2.0 (1.5-2.8)**	**<0.001**	**1.7 (1.2-2.5)**	**0.0024**

Model 1: adjusted for age, sex, and education.
Model 2: model 1 adjusted for smoking, alcohol, physical activity, heart disease, neoplasms, diabetes, CKD, MSK diseases, and COPD. Model 2 excluded 10 participants due to missing data on smoking and/or alcohol consumption.
Abbreviations: CI = confidential interval; COPD = chronic obstructive pulmonary disease; CKD = chronic kidney disease; GDF-15 = growth differentiation factor 15; MSK = musculoskeletal; OR = odds ratio

Supplementary Table 4. Association between GDF-15 and sarcopenia status at baseline excluding participants with musculoskeletal diseases at baseline (n = 458)

GDF-15 (ng/mL)	Model 1<br>OR (95% CI)	p-value	Model 2<br>OR (95% CI)	p-value
Non sarcopenic	Ref	-	Ref	-
Probable sarcopenic	**1.5 (1.1-1.9)**	**0.0024**	**1.4 (1.06-1.8)**	**0.0172**
Sarcopenic	**1.8 (1.3-2.6)**	**<0.001**	1.4 (0.96-2.1)	0.0769

Model 1: adjusted for age, sex, and education.
Model 2: model 1 adjusted for smoking, alcohol, physical activity, heart disease, cerebrovascular diseases, diabetes, CKD, neoplasms, and COPD. Model 2 excluded 8 participants due to missing data on smoking and/or alcohol consumption.
Abbreviations: CI = confidential interval; COPD = chronic obstructive pulmonary disease; CKD = chronic kidney disease; GDF-15 = growth differentiation factor 15; OR = odds ratio

Supplementary Table 5. Association between GDF-15 and risk of belonging to the early progression trajectory of sarcopenia excluding participants with neoplasms at baseline (n = 194)

GDF-15 (ng/mL)	Model 1 OR (95% CI)	p-value	Model 2 OR (95% CI)	p-value
**Continuous**	**1.7 (1.4-2.1)**	**<0.001**	**1.5 (1.2-1.8)**	**<0.001**
**1st quintiles**	Ref	-	Ref	-
**2nd quintiles**	1.05 (0.80-1.38)	0.7347	1.04 (0.79-1.37)	0.7883
**3rd quintiles**	1.10 (0.84-1.46)	0.4804	1.08 (0.82-1.43)	0.5812
**4th quintiles**	**1.58 (1.19-2.10)**	**0.0017**	**1.41 (1.05-1.89)**	**0.0235**
**5th quintiles**	**1.92 (1.45-2.75)**	**<0.001**	**1.58 (1.12-2.22)**	**0.0084**

Model 1: adjusted for age, sex, and education.
Model 2: model 1 adjusted for smoking, alcohol, physical activity, heart disease, cerebrovascular diseases, diabetes, CKD, MSK diseases, and COPD. Model 2 excluded 9 participants due to missing data on smoking and/or alcohol consumption.
Abbreviations: CI = confidential interval; COPD = chronic obstructive pulmonary disease; CKD = chronic kidney disease; GDF-15 = growth differentiation factor 15; MSK = musculoskeletal; OR = odds ratio

Supplementary Table 6. Association between GDF-15 and risk of belonging to the early progression trajectory of sarcopenia excluding participants with cerebrovascular diseases at baseline (n = 129)

GDF-15 (ng/mL)	Model 1 OR (95% CI)	p-value	Model 2 OR (95% CI)	p-value
**Continuous**	**1.8 (1.4-2.2)**	**<0.001**	**1.6 (1.3-1.9)**	**<0.001**
**1st quintiles**	Ref	-	Ref	-
**2nd quintiles**	1.06 (0.81-1.39)	0.6497	1.05 (0.80-1.38)	0.7354
**3rd quintiles**	1.14 (0.87-1.49)	0.3545	1.11 (0.84-1.46)	0.4579
**4th quintiles**	**1.54 (1.16-2.04)**	**0.00257**	**1.38 (1.03-1.85)**	**0.0286**
**5th quintiles**	**1.96 (1.43-2.68)**	**<0.001**	**1.63 (1.17-2.27)**	**0.0041**

Model 1: adjusted for age, sex, and education.
Model 2: model 1 adjusted for smoking, alcohol, physical activity, heart disease, neoplasms, diabetes, CKD, MSK diseases, and COPD. Model 2 excluded 10 participants due to missing data on smoking and/or alcohol consumption.
Abbreviations: CI = confidential interval; COPD = chronic obstructive pulmonary disease; CKD = chronic kidney disease; GDF-15 = growth differentiation factor 15; MSK = musculoskeletal; OR = odds ratio

Supplementary Table 7. Association between GDF-15 and risk of belonging to the early progression trajectory of sarcopenia excluding participants with musculoskeletal diseases at baseline (n = 458)

GDF-15 (ng/mL)	Model 1<br>OR (95% CI)	p-value	Model 2<br>OR (95% CI)	p-value
**Continuous**	**1.6 (1.3-2.0)**	**<0.001**	**1.4 (1.1-1.7)**	**0.0039**
**1st quintiles**	Ref	-	Ref	-
**2nd quintiles**	1.01 (0.76-1.35)	0.9467	1.00 (0.74-1.34)	0.9797
**3rd quintiles**	1.07 (0.79-1.43)	0.6747	1.05 (0.78-1.42)	0.7503
**4th quintiles**	1.35 (1.00-1.82)	0.0518	1.20 (0.88-1.65)	0.2422
**5th quintiles**	**1.81 (1.29-2.54)**	**<0.001**	**1.51 (1.05-2.17)**	**0.0255**

Model 1: adjusted for age, sex, and education.
Model 2: model 1 adjusted for smoking, alcohol, physical activity, heart disease, neoplasms, diabetes, CKD, cerebrovascular diseases, and COPD. Model 2 excluded 8 participants due to missing data on smoking and/or alcohol consumption.
Abbreviations: CI = confidential interval; COPD = chronic obstructive pulmonary disease; CKD = chronic kidney disease; GDF-15 = growth differentiation factor 15; OR = odds ratio.
